# Second-Harmonic Enhancement from a Nonlinear Plasmonic Metasurface Coupled to an Optical Waveguide

**DOI:** 10.1021/acs.nanolett.1c04584

**Published:** 2022-04-03

**Authors:** Tsafrir Abir, Mai Tal, Tal Ellenbogen

**Affiliations:** † Department of Condensed Matter Physics, School of Physics and Astronomy, Tel Aviv University, Tel Aviv 6779801, Israel; ‡ Department of Physical Electronics, School of Electrical Engineering, Tel-Aviv University, Tel Aviv 6779801, Israel; § Center for Light-Matter Interaction, Tel-Aviv University, Tel Aviv 6779801, Israel

**Keywords:** metasurface, waveguide, nonlinear, collective scattering, guided-mode resonance, guided lattice resonance

## Abstract

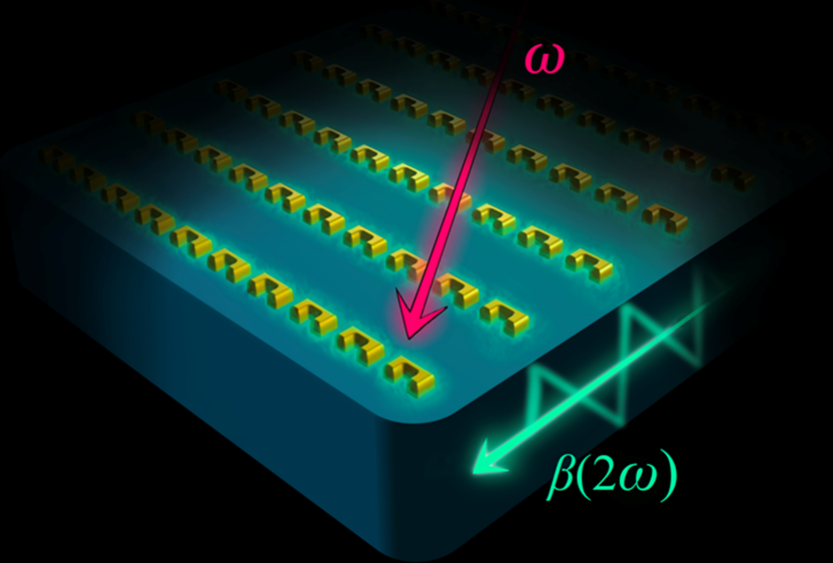

Metasurfaces are commonly constructed from two-dimensional arrangements of nanoresonators. Coherent coupling of the nanoresonators through extended photonic modes of the metasurface results in a modified collective optical response, and enhances light–matter interactions. Here we experimentally demonstrate that strong collective resonances can arise also from coupling the metasurface to an optical waveguide. We explore the effect this waveguide-assisted collective interaction has on second-harmonic generation from the hybrid system. Our measurements indicate an enhancement factor of 8 for the transmitted second harmonic in comparison to incoherent collective scattering. In addition, complementary simulations predict about a 100-fold enhancement for the second harmonic that remains confined inside the waveguide. The ability to control the hybrid modes by the waveguide’s design provides broader control over the formation of the collective interaction and new tools to tailor the nonlinear interactions. Our findings pave a promising direction to realize nonlinear photonic circuits with metasurfaces.

In recent years much effort has been devoted to the research of nonlinear optical metamaterials and nonlinear metasurfaces.^1,2^ Various frequency conversion processes utilizing nonlinear metasurfaces have been reported, including second-^3^ to high-harmonic^4^ generation. Also, difference frequency generation processes were shown to yield terahertz radiation,^5^ for example, and even to generate entangled photon pairs.^6^ Moreover, the ability to control the optical response on a microscopic scale through a precise design of the metasurface provides means to modulate the nonlinear wavefront.^7−9^ Although metasurfaces were found to be compelling compact and versatile platforms for tailored nonlinear optical interactions, low total conversion efficiencies hinder their adoption for technological applications.

In general, the optical response of a metasurface is determined by both the single nanoresonator’s properties and the collective interactions between different lattice sites. When the lattice spacing is approaching the effective wavelength in the host medium, resonant collective scattering between lattice sites dramatically alters the optical response of the array. This is known in the literature as a Rayleigh–Wood anomaly (RA),^10^ which is when a diffraction order is on the edge between a radiating to an evanescent mode. This type of optical anomaly is associated with sharp spectral features, as the collective scattered fields coherently build into a surface wave. In a metasurface made out of resonant nanoantennas, the localized modes and the distributed surface mode at the RA condition can hybridize, to form a surface lattice resonance (SLR).^11^ These hybrid modes were found to significantly enhance nonlinear wave mixing.^12−16^ Yet, to fully harness the potential of these modes, the metasurface needs to be placed in a homogeneous dielectric background.^17^ When the integration of metasurfaces in compact optical systems is considered, scattered fields from inhomogeneities reduce the quality of the RAs. This imposes some limitations and restrictions for incorporating collective scattering effects in metasurface integrated systems, such as photonic circuits.

When a diffractive periodic array is in optical contact with a waveguiding structure, diffraction orders may couple to guided modes.^18^ This coupling results in sharp spectral features associated with the leaky modes known as guided mode resonances (GMRs).^19^ For arrays of subwavelength scatterers, this means coherent scattering that is mediated by the guided modes. While gratings have been extensively used to couple light in and out of waveguides, the interactions between localized modes in metasurface and propagating GMRs have been left relatively unexplored.^20,21^ Such interactions lead to mode hybridization^22^ similar to the SLRs found when localized modes are coupled to RAs. In the literature these hybrid modes are sometimes referred to as waveguide-plasmon polaritons.^23^ Since this collective phenomenon is not unique to plasmonics, we prefer to use the more general term guided lattice resonance (GLR) instead. GLRs were found to enhance fluorescence^24,25^ and even stimulate lasing.^26^ GMRs by themselves were reported to enhance nonlinear optical interactions, such as second-^27,28^ and third-harmonic^29^ generation, or to achieve ultrahigh-quality linear and nonlinear metasurfaces.^30,31^ However, in these works the nonlinearity originated from the susceptibility of the waveguide’s bulk media and from surface effects, but not from the metasurface’s nonlinearity. Here, we study experimentally and numerically the effect of nonlinear GLRs on the enhancement of second-harmonic generation (SHG) emitted to free space and confined in the optical waveguide. Additionally, we show how these modes, in contrast to SLRs, are insensitive to the requirement for a homogeneous refractive index background.

When a plane wave is incident upon the metasurface the diffraction orders are determined by the conservation of parallel momentum
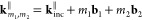
1where **b**_1,2_ are the primitive reciprocal lattice vectors, *m*_1,2_ are integers, **k**_inc_ and **k**_*m*1,*m*2_ are the incident and the diffracted wave vectors, respectively, and the superscript ∥ stands for the vector’s projection on the metasurface plane. If the metasurface is in optical contact with a planar waveguide, a GMR is formed when
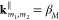
2where β_*M*_ is the *M*th-order guided mode’s propagation constant. This condition is schematically described by the arrows in Figure 1a. For a waveguiding slab, finding β_*M*_ requires solving transcendental equations for both the transverse electric (TE) and transverse magnetic (TM) polarizations^32^
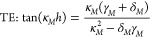
3
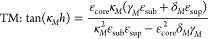
4where , , , *h* is the slab’s thickness, *k*_0_ is the wavenumber in vacuum, and ε_core_, ε_sub_, and ε_sup_ are the permittivities of the core, substrate, and superstate, respectively. Each guided mode may couple to multiple diffraction orders, resulting in a large number of supported GMRs. The coherent scattering at the metasurface, together with the near-field enhancement associated with guided modes can be beneficial for nonlinear wave-mixing processes such as SHG. In general, the nonlinearities may originate from each of the hybridized system’s constituents. However, in this work we focus on the case where the quadratic nonlinearity originates from the metasurface.

**Figure 1:**
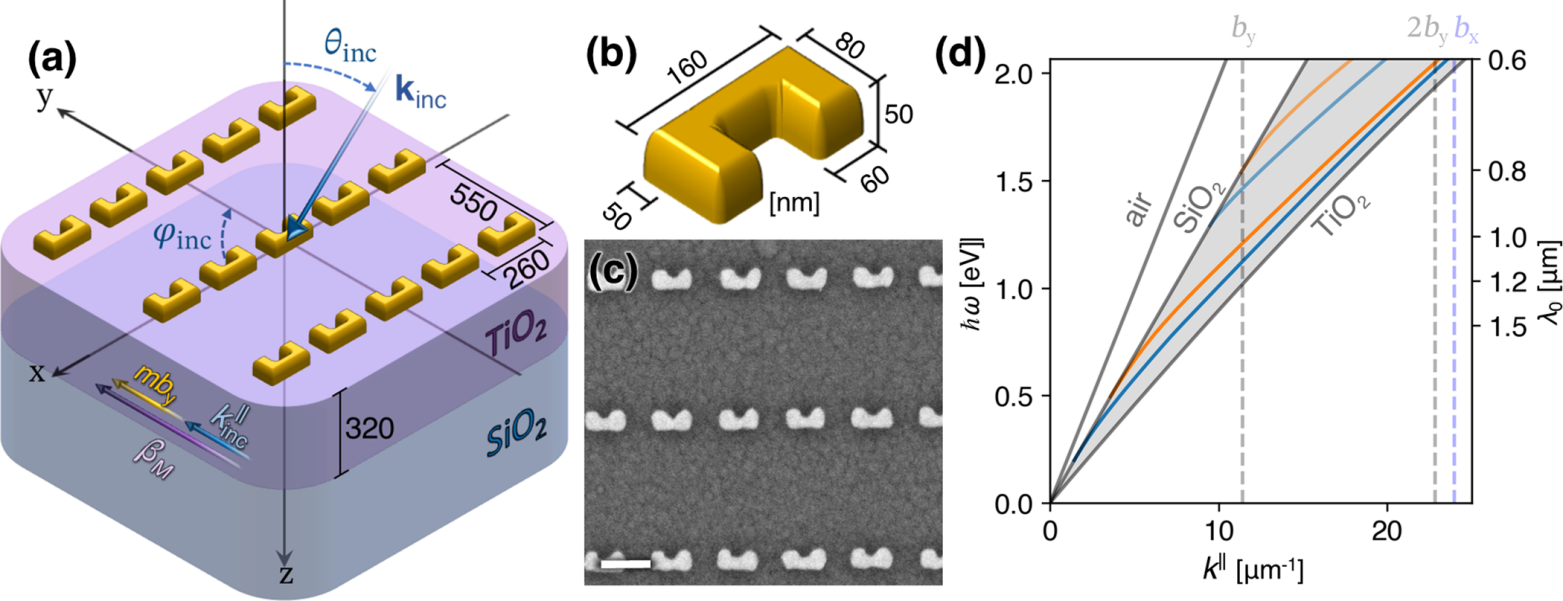
Metasurface–waveguide hybrid system description. (a) Schematic description of the plasmonic metasurface on top of a TiO_2_ planar waveguiding layer. Numeric parameters are given in nanometers. φ_inc_ and θ_inc_ are the azimuthal and polar angles of the incident field, respectively. (b) Illustration depicting the mean dimensions of the gold SRRs. Values are given in nanometers. (c) SEM image of the fabricated plasmonic metasurface on top of the thin TiO_2_ layer. The white scalebar is 200 nm. (d) Dispersion of the guided modes. Blue and orange lines show the dispersion of the first two guided modes for both TE and TM modes, respectively. The dashed lines mark the reciprocal lattice vectors, and the gray diagonals are the light lines of the substrate, core, and superstrate.

The studied system of a metasurface–waveguide hybrid is schematically described in Figure 1a. The waveguiding layer is made of a 320 nm thick TiO_2_ film, sputtered on a fused silica substrate. A 100 × 100 μm^2^ metasurface of gold split-ring resonators (SRRs) was fabricated on top of the waveguide by a conventional e-beam lithography technique. Figure 1a,b illustrates the SRRs’ shape, dimensions, and lattice spacing and Figure 1c presents a scanning electron microscope (SEM) image of the metasurface. The TiO_2_ surface roughness led to some irregularities of the fabricated SRRs’ shape; thus, the dimensions presented in Figure 1b are the typical mean values measured from multiple scanning electron microscope images. The meta-atoms were fabricated in a rectangular lattice, with interparticle spacings *a*_*y*_ = 550 nm and *a*_*x*_ = 260 nm, so as to support diffraction in *y* and suppress the diffraction in *x*. The guided modes’ dispersions were evaluated using eqs 3 and 4 with the substrate’s refractive index taken from the literature and the evaluated refractive index of the sputtered TiO_2_ (see Supporting Information). The light lines and the modes’ dispersion are presented in Figure 1d, where the reciprocal lattice vectors are marked by dashed vertical lines. The intersections of these lines with the guided modes’ dispersion and light lines represent the GMR and RA conditions, respectively. By taking oblique incidence angles, parallel momentum is added/subtracted to shift these intersections and provide the means to spectrally tune the GMR. Noncentrosymmetric SRRs were chosen for the metasurface elements, as they were shown to support strong quadratic nonlinearities and have already been investigated extensively in the context of SHG.^3,7,9,33^ When the pump is polarized parallel to the base of the SRR, it excites the LSPR at the fundamental frequency (FF) and the generated second-harmonic (SH) is predominantly orthogonally polarized, parallel to the SRR’s arms.^34^ This cross-polarization behavior is an indication that the SH originates from the metasurface and not from the dielectric interfaces of the waveguiding stratified structure. The resonators’ dimensions can be adjusted to tune the LSPRs to the frequencies of interest and benefit from near-field enhancement to promote the nonlinear wave-mixing.

The first step in characterizing the hybrid system was to perform angle- and polarization-resolved linear transmission measurements (see the Supporting Information). The azimuthal angle φ_inc_ was kept constant at 90°, while θ_inc_ was varied to sweep over the parallel momentum **k**_inc_^∥^ = *k*_0_ sin θ_inc_*ŷ*. The measured transmission spectra are presented in Figure 2, along with the calculated, angle-dependent GMR dispersion. Each mode is labeled by a subscript marking the guided modes’ order and a superscript labeling the diffraction order in *ŷ* (the diffraction order in *x̂* is zero, and its labeling was omitted for brevity). The polarization-dependent dispersion of the GMRs is evident from the transmission, as each of the transmission spectra in Figure 2 exhibit different GMR-related spectral features. Additionally, we may notice how TE or TM GMRs interact with different LSPR modes. GLRs are formed when the GMR spectrally overlaps with a LSPR, which are manifested by the splitting of the transmission dips. As expected, the high refractive-index contrast between the waveguide’s core and the superstrate diminish RA-related features in the transmission spectra.^17^

**Figure 2:**
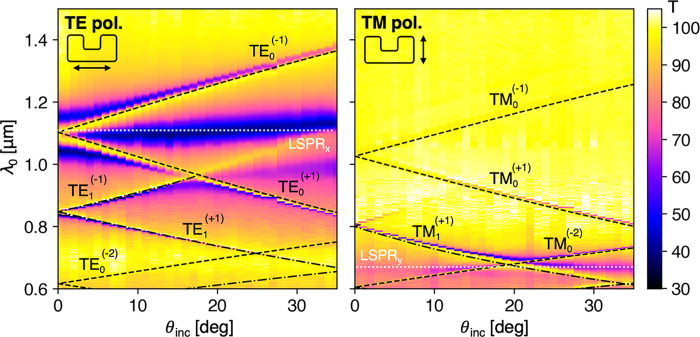
Angle- and polarization-resolved transmission measurements where the reference measurements are made from a region with no metasurface. The left and right panels show the transmission for the TE and TM polarizations, respectively. The black dashed lines show the GMRs of zeroth-order guided modes and the dotted-dashed lines those of the first guided mode. The GMRs were labeled by the guided mode’s order (subscript) and polarization and the diffraction order (superscript). The white dotted lines mark the LSPRs excited in SRRs for each polarization. Values slightly exceeding 100% in the TM polarization are due to some random spectral noise in the illumination source.

To characterize the way the GLRs affect the SHG, the sample was pumped in TE polarization by a tunable femtosecond laser with an average power of 200–300 mW and pulse length of 140 fs (see Supporting Information for additional details). The measured average SH photon counts were corrected by the quantum efficiency of the detector and were normalized by the square of the pump’s power. Figure 3a presents the TM-polarized SH measured at the zeroth-order transmission. The dispersions of the GMRs were added with the TM modes corresponding to SH wavelengths at λ_pump_/2, while the TE mode corresponds to λ_pump_. The region of maximum SHG follows the dispersion of the TE_0_^(−1)^ GMR, which means that it is predominantly enhanced by resonances at the FF. When the angle increases, the resonance for the FF red-shifts to better overlap with the LSPR of the SH. This overlap leads to an estimated enhancement factor of 8, relative to the results obtained at normal incidence, where the GMRs are distant and barely contribute. These findings are comparable to the reports for case of RA SLRs at the pump frequencies.^15^ Additionally, GMR features related to SH frequency, i.e. the TM modes, appear as dips that indicate the coupling of the generated SH to the waveguide.

**Figure 3:**
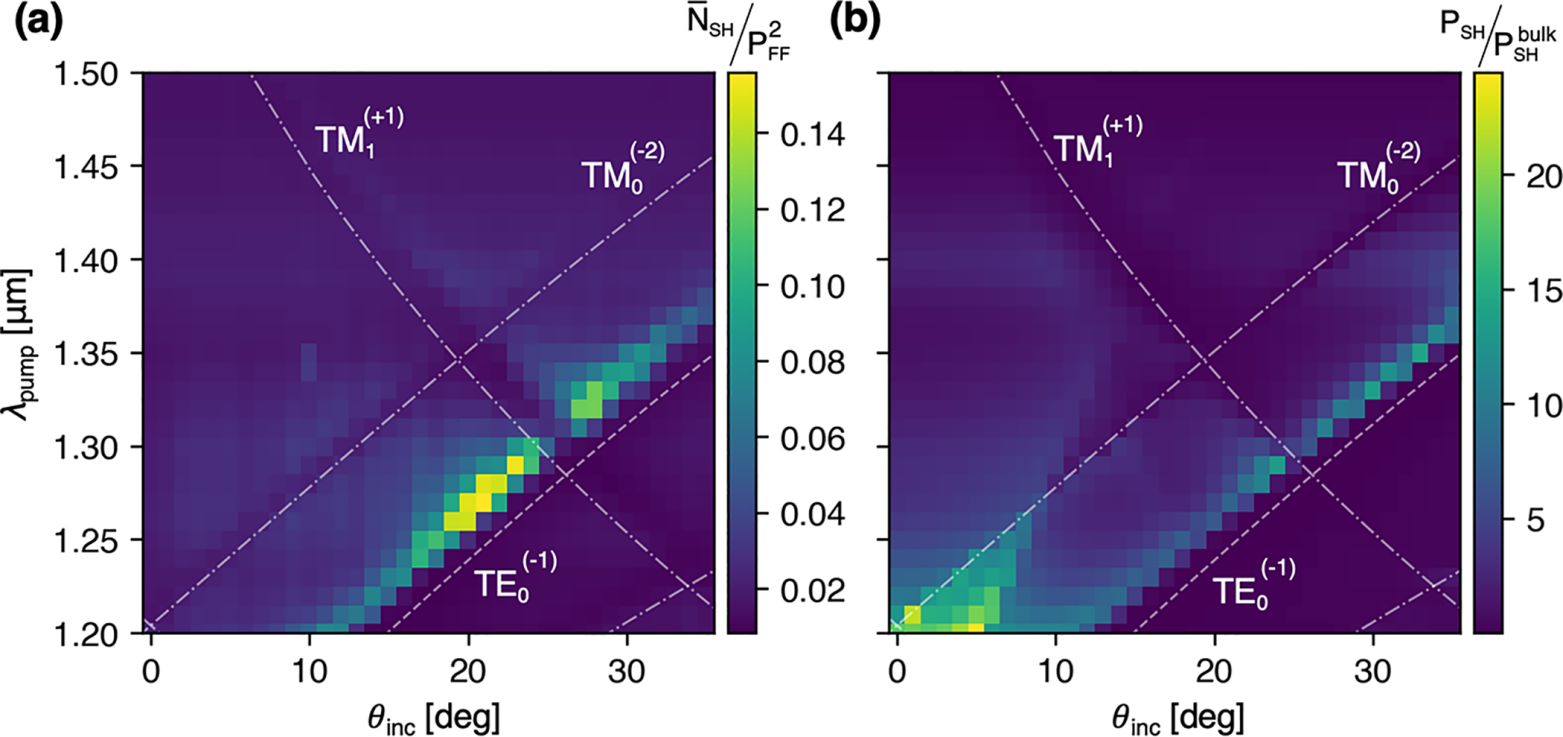
Transmitted second harmonic. (a) Measured transmitted SH as a function of the pump’s incident angle and wavelength. The measured photon count was normalized by the pump power squared. (b) Simulated transmitted SH normalized by the total SH from the same metasurface without the waveguide, to give an evaluation of the SHG enhancement. The white dashed lines represent the dispersion of the TE GMRs. The dotted-dashed lines represent the dispersion of the TM GMR modes corresponding to the SH wavelength λ_pump_/2.

To gain a better understanding of the nonlinear dynamics, we performed full-wave simulations using a commercial solver with the hydrodynamic model as the source of the nonlinear harmonic generation.^35^ From the simulation at the frequency of the pump (ω) the linear polarization (**P**_1_) can be found. Using the hydrodynamic model, the relation between the induced nonlinear surface currents (**K**_NL_) in the plasmonic nanoresonators to the linear polarization is approximated by
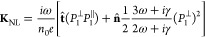
5where *n*_0_ = 5.7 × 10^28^ m^–3^ is the electron density, γ = 1.07 × 10^14^ s^–1^ is the phenomenological damping rate, and **t̂** and **n̂** are the unit vectors pointing parallel and normal to the metallic surface, respectively. Similarly, superscripts of *P*_1_ indicate the polarization component perpendicular and parallel to the metallic surface. These nonlinear currents serve as the radiation source for the simulation at the SH frequency. To evaluate the enhancement factor, the transmitted SH in the simulation was normalized by the results obtained for the same metasurface on a semi-infinite TiO_2_ substrate. In the simulations, periodic boundary conditions defined a unit cell that follows the lattice spacing mentioned in Figure 1a. The SRR, with the dimensions mentioned in Figure 1b, was positioned in the center of the unit cell. The resulting LSPRs were red-shifted by about 30 nm in comparison to those in the measurements; these led to some deviations of the SHG features presented in Figure 3b. The enhancement seen near λ_pump_ = 1.2 μm at small angles is related to the red-shift of the GLR at λ_pump_. The steep spectral feature starting at ∼5° is the free space RA at the superstrate. It is not captured in the experiment due to the imperfections of the fabricated waveguide and SRRs. Other RA-related features do not appear at all, as expected due to the high refractive index contrast at the metasurface’s plane. The overall resemblance to the experimental results validated the simulations, which provided us with the means to probe the near fields.

We used the simulations to qualitatively study how the collective interaction affects the SHG coupled to the waveguide. Figure 4a shows the normalized SHG power confined to the waveguide as a function of incidence angle and wavelength. Since the SH is coupled through the diffraction orders, it can couple to counterpropagating TM modes. Therefore, the sign and color in Figure 4a indicate the direction of the power flow. It can be seen how the enhancement factor may reach up to 2 orders of magnitude. The upper panel shows a cross-section at of this enhancement at λ_pump_ = 1.29 μm. Figure 4b reveals the normalized TM field (*H*_*x*_) of the generated SH in the unit cell for λ_pump_ = 1.32 μm and at  for two different angles (stated at the panels’ upper right corners). The field profiles match the familiar mode profiles from guided-mode theory and validate how SHG feeds the TM modes. Overall they demonstrate how coupling of the SHG to the GMRs leads to an enhancement inside the waveguide by 2 orders of magnitude, which is 1 order of magnitude larger than the SH emitted to free space by the same system and also exceeds those in reports for the enhancement obtained from RA-based SLRs.^13,15^ Additionally, the guided mode profiles, described by the white lines in Figure 4b, reveal how the coupling of nonlinear metasurface was obtained by placing it at the evanescent tail of the guided modes. A thoughtful design, in which the metasurface is better positioned relative to the guided modes’ profile, may lead to an even stronger enhancement of the nonlinear emission into the waveguide.

**Figure 4:**
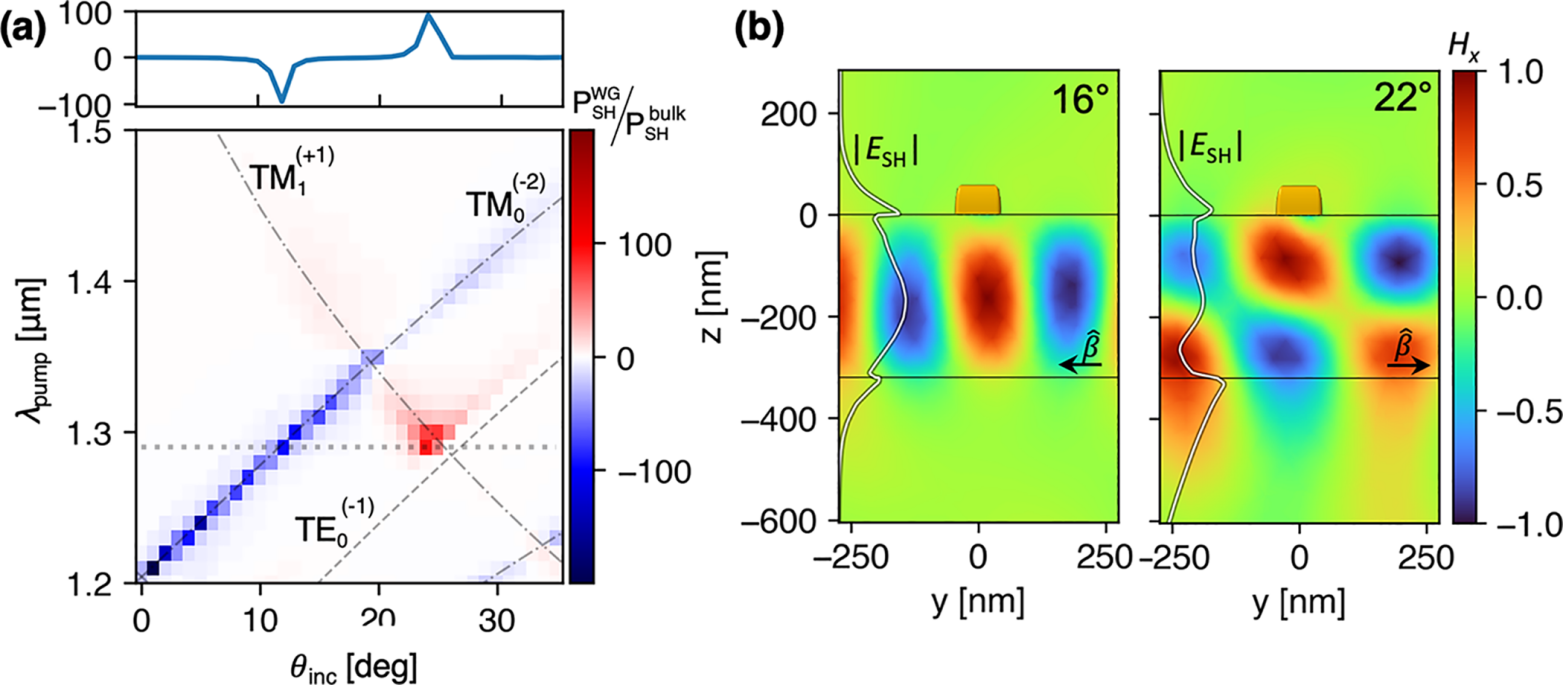
Second-harmonic near fields. (a) Simulation results for the SH power flow confined in the waveguide normalized by the total emitted SH from the same metasurface without the waveguide, to provide an evaluation of SHG enhancement obtained when it is coupled to GMRs. The sign signifies the power flow direction in *y*. The upper panel is a cross-section at λ_pump_ = 1.29 μm and is indicated by the dotted line in the lower panel. (b) TM mode profiles in the unit cell at *x* = *a*_*x*_/2, λ_SH_ = 0.66 μm, and θ_inc_ = 16°/22°. The magnetic fields were normalized to 1, and the white line on the left qualitatively illustrates the mode’s profile in terms of the electric field magnitude |*E*_SH_|. β̂ with an arrow indicates the propagation direction of the guided mode, and the black lines frame the waveguide’s core with the SRR’s location marked by the yellow rectangle at the top representing the side view of the SRR’s arm.

To conclude, we have demonstrated how a metasurface in optical contact with a planar waveguide has additional channels available to achieve coherent scattering between lattice sites. This occurs through the coupling of the metasurface’s diffraction orders to the guided modes. When these GMRs spectrally overlap with LSPRs, it results in polarization-dependent GLRs. These, in contrast to the RA-based SLRs, are insensitive to index matching and do not require a homogeneous dielectric environment. The GLRs provide similar enhancements of the SH emitted to free space, in comparison to SLRs. Moreover, simulations predict an additional order of magnitude increase in the enhancement of the SH confined to the waveguide. This enhancement may be attributed to two mechanisms. The first is the increase in effective polarizability of the nonlinear SRRs due to the collective resonances. The second is the near-field enhancement in the vicinity of the waveguide upon excitation of a guided mode^29^ that can occur for both the FF and the SH. The increase in effective polarizability and the stronger near fields enhance the light–matter interactions and the nonlinear conversion process. On the basis of these results, together with the large number of design degrees of freedom in the hybrid system, even higher enhancements in the wave-mixing conversion process may be achieved. Eventually, combining nonlinear metasurfaces with optical waveguides provides new means to infuse future photonic devices with nonlinear interactions.

## Supporting Information Available

Refractive indices used for both the calculation of the guided modes’ dispersion and simulations and details and description of the experimental setups and methods used for the linear and second-harmonic measurements (PDF)
