# Supplementary material for: Second-Harmonic Enhancement from a Nonlinear Plasmonic Metasurface Coupled to an Optical Waveguide
Source: Nano Lett. 2022 Apr 3;22(7):2712–7. doi: 10.1021/acs.nanolett.1c04584 (PMC9011386; doi:10.1021/acs.nanolett.1c04584)
Supplement: Supplementary file 1 — nl1c04584_si_001.pdf [file nl1c04584_si_001.pdf]

Supporting Information

Second harmonic enhancement from a nonlinear plasmonic metasurface coupled to an optical waveguide

Tsafrir Abir[†,‡,§], *Mai Tal*[†,‡,§] *& Tal Ellenbogen*[‡,§]

[†] Department of Condensed Matter Physics, School of Physics and Astronomy, Tel Aviv University, Tel Aviv 6779801, Israel

[‡] Department of Physical Electronics, School of Electrical Engineering, Tel-Aviv University, Tel Aviv 6779801, Israel

[§]Center for Light-Matter Interaction, Tel-Aviv University, Tel Aviv 6779801, Israel

This document contains supplementary information regarding the refractive index used for the calculation of the guided modes' dispersion and in the simulations. Additionally, it provides description of the experimental setup and methods used for the linear and second-harmonic measurements.

1. Waveguide preparation and characterization

Initially, cut slides of fused silica were cleaned with O_2 plasma, sonicated in acetone, washed with IPA and dried using a N_2 gun. Then a ~300 nm layer of TiO_2 was deposited using a standard magnetron sputterer. A clean silicon substrate with a thermal oxide layer was also placed in a chamber of the sputtering machine to serve in the ellipsometry measurements. The thickness and the refractive index of the TiO_2 film were measured using Woollam Spectroscopic Ellipsometers M-2000D. The data from the ellipsometer was fitted using the ellipsometer's software to a Cauchy equation. The results are presented in Figure S*1* and the resulting thickness was 310±20 nm. The SiO_2 refractive index was taken from the literature[1], and is also shown for comparison. The optical properties of the gold SRRs were interpolated from the results of Johnson and Christy[2] .

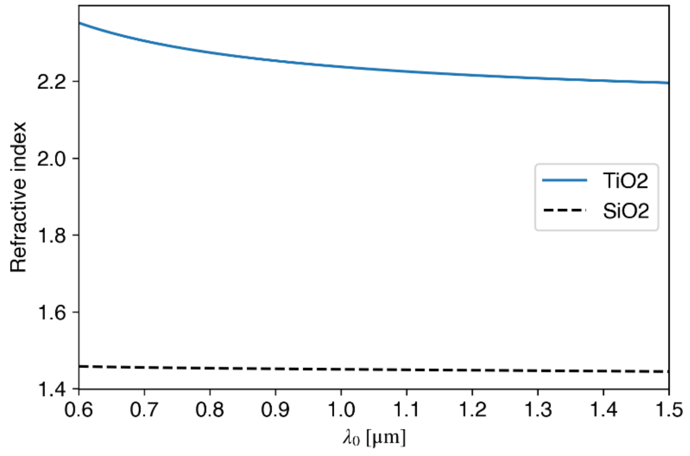

Figure S1 – Refractive index of the waveguiding structure's materials: sputtered TiO_2 and SiO_2.

2. Experimental setup and measurements

2.1. Linear transmission measurements

To characterize the angle dependent dispersion of the fabricated sample the sample was illuminated by a supercontinuum laser (NKT photonics Super-K compact), and transmission was measured. The TM and TE polarization were interchanged using a polarizer (see Figure S2). The sample was mounted on a rotating stage on top of a XYZ stage. This provided us with the ability to perform measurements on a specific part of the sample for varying angles of incidence θ_{inc}. The light passing through the sample has been collected using Mitutoyo Apochromatic 50X Objective. Since the objective is infinity corrected an additional tube lens with f=200 mm was used. The image is then duplicated into two using a beam-splitter, where one image passed to CCD camera to visualize the part of the sample being measured and make the measurement process more convenient. The other image was directed onto an iris to selectively pass only a small region (either metasurface or reference) to be measured. The light that passed through the iris was focused into either one of two fibers. One fiber fed Andor's Shamrock spectrograph with Newton EMCCD detector to measure the wavelength range 600-950 nm. The second fiber was used to measure the range between 900-1500 nm with Ocean Optics NIR Quest. The spectral overlap between the two spectrometer ensures smooth stitching of the spectra. Switching the optical path between the two spectrometers was done by a flip mirror.

At each polarization and angle, a new reference measurement was taken by moving the sample so only the part with the stratified structure is measured (no metasurface). This has been done for both spectrometers. Performing these type of adjusted transmission measurements provided us with the means to observe the effect of the metasurface and the coupling to the waveguide.

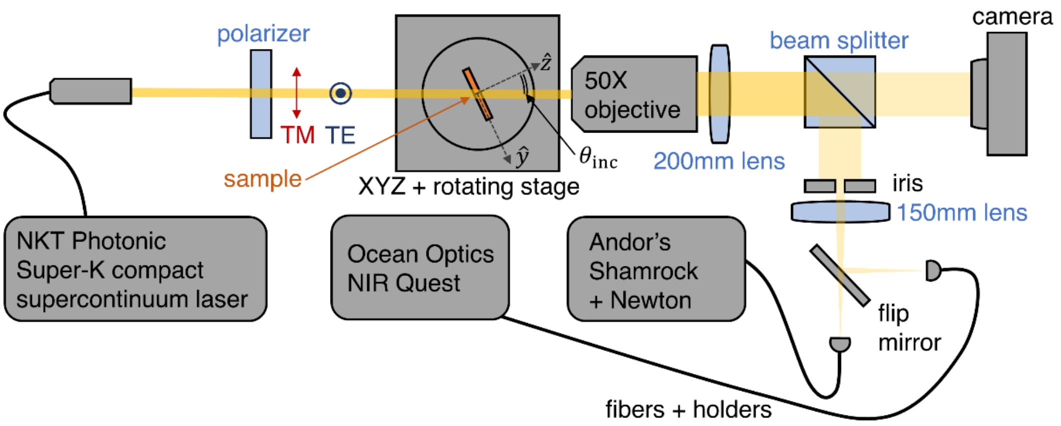

Figure S2 - Schematic description of the optical setup used for the transmission measurements.

2.2. Transmitted second harmonic measurement

The pump beam originated from Coherent's Chameleon and Compact OPO system. The system outputs ~140 fs pulses at a repetition rate of 80 MHz, with the OPO providing the means to tune the frequency of the pump. The pump was directed through a half wave-plate and a polarizer to control the polarization and power of the beam reaching the sample. The experiment was performed with the pump at TE polarization (see Figure S2). The beam was focused by an achromatic doublet lens with a focal length of 200 mm. Before the sample, a 1000 nm longpass filter was used to block any parasitic signals and a beam splitter was placed to send part of the pump to an Ophir NOVA II power meter. Calibration measurements were made to correlate between the power at the reflected and transmitted arms of the

beam splitter for different wavelengths. The illuminating beam was weakly focused and the power reaching the sample was kept in the range of 200-300 mW, to avoid causing damage to the metasurface. The 0th order transmitted pump beam and the generated second harmonic were collected by the objective lens where two 1000 nm shortpass filters blocked the pump to allow only the second harmonic to reach the sensitive scientific CMOS camera (Hamamatsu Orca Flash 4.0). A tube was used to reduce the amount of environmental scattered light that reaches the detector. The pixels in the spot's area of the nonlinear image were averaged and corrected by camera's quantum efficiency and the square of the pump's power. This type of measurements has been performed for the range of the pump frequencies and angles represented in Figure 3a (in the main text).

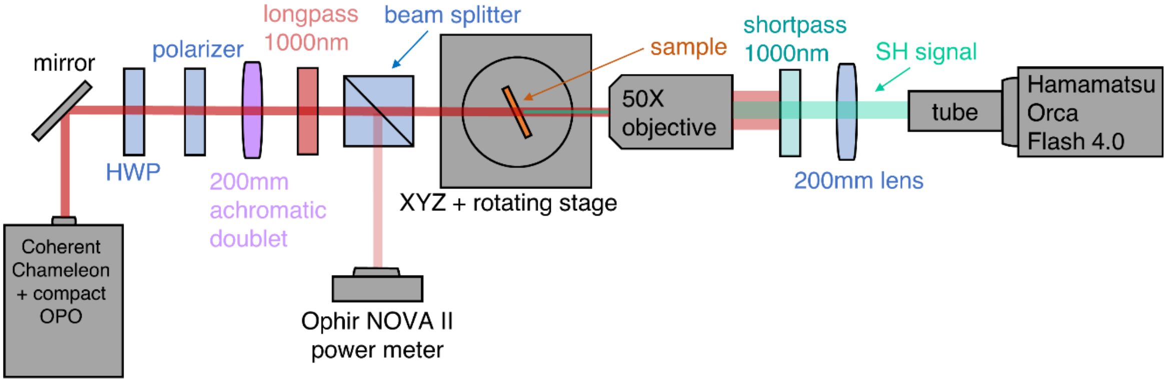

Figure S3 – Schematic description of the optical setup used for the second-harmonic measurements.

References

(1) Malitson, I. H. Interspecimen Comparison of the Refractive Index of Fused Silica. *J. Opt. Soc. Am.* **1965**, *55*, 1205.

(2) Johnson, P. B.; Christy, R. W. Optical Constants of the Noble Metals. *Phys. Rev. B* **1972**, *6*, 4370–4379.
